# Effect of operators’ proficiency level and patients’ related factors on possible complications, using a high frequency polyamide sonic intracanal irrigation device: A prospective clinical cohort study

**DOI:** 10.1371/journal.pone.0285492

**Published:** 2023-05-04

**Authors:** Tobias Hahn, David W. Christofzik, Karim Fawzy El-Sayed, Sandra Freitag-Wolf, Jonas Conrad, Christian Graetz, Birte Größner-Schreiber, Christof Dörfer

**Affiliations:** 1 Clinic for Conservative Dentistry and Periodontology, Christian-Albrechts-University of Kiel, Kiel, Germany; 2 Faculty of Oral and Dental Medicine, Oral Medicine and Periodontology Department, Cairo University, Cairo, Egypt; 3 Institute of Medical Informatics and Statistics, Christian-Albrechts-University of Kiel, University Hospital Schleswig-Holstein Campus Kiel, Kiel, Germany; Yerevan State Medical University Named after Mkhitar Heratsi, ARMENIA

## Abstract

**Objectives:**

Sonic/ultrasonic devices are essential tools in today’s endodontics. This prospective trial evaluated for the first time the impact of practitioners’ proficiency levels and patient-related factors on complications associated with a high frequency polyamide sonic irrigant activation device.

**Methods:**

In total 334 patients (females:158, males:176; age:18–95 years) received in the course of their endodontic therapy an intracanal irrigation, using a high frequency polyamide sonic irrigant activation device, by practitioners of different proficiency levels (undergraduate students, general practitioners or endodontists). Intracanal bleeding (yes/no), postoperative pain (0–10 scale), emphysema (yes/no) and polyamide tip fractures (yes/no) were recorded and related to proficiency levels, age, gender, tooth type, smoking-status, systemic conditions affecting healing ability, baseline pain, swelling, fistula, sensitivity to percussion and diagnosis.

**Results:**

Intracanal bleeding was associated with patients’ age (p<0.05), baseline pain level (OR = 1.14, 95%CI = 0.91–1.22) and baseline swelling (OR = 2.73, 95%CI = 0.14–0.99; p<0.05) but not proficiency level, gender, tooth type, smoking, systemic conditions, baseline fistula or sensitivity to percussion (p>0.05). Postoperative pain development was related to proficiency level (p<0.05) and baseline pain level (p<0.001), with no influence of age, gender, tooth type, smoking, systemic conditions, baseline fistula, swelling or sensitivity to percussion (p>0.05). Emphysema and polyamide tip fractures were not reported.

**Conclusions:**

Within the current study’s limitations, younger patients with higher baseline pain and swelling, were associated with higher intracanal bleeding. Apart from higher postoperative pain observed with less experienced practitioners, proficiency level had no influence on bleeding, polyamide tip fracture or emphysema, endorsing the high frequency polyamide sonic irrigation device as a safe therapeutic device.

## Introduction

Root canal treatment involves the three-dimensional cleaning and shaping of the root canal system to be obturated by an inert bio-compatible filling material [1]. Yet, the diversity in root canal anatomies makes it impossible to adequately clean the root canal system, relying solely on mechanical instrumentation [2]. This necessitates the additional usage of chemical root canal irrigants for the removal of inflamed and infected pulpal tissue remnants, planktonic microorganisms, bacterial biofilms, smear layer as well as for the effective extirpation of mechanically produced dentine debris [3]. The efficiency of this chemo-mechanical approach in turn affects the long-term success of root canal treatment outcomes [4].

Although, sodium hypochlorite (NaOCl) is currently one of the most widely used intracanal irrigation solutions, its major drawbacks remains to be its high surface tension, possibly affecting its ability for tubular penetration and microbial cytotoxicity [5]. In this context irrigants’ activation/agitation strategies, using sonic (160 Hz–6 kHz) and ultrasonic (25–30 kHz) devices, were introduced to generate flow and shear stresses within the intracanal irrigant fluids [6] and thereby improve the efficiency of NaOCl in eliminating of intra-canal microorganisms by up to 90% [7]. A high frequency polyamide sonic irrigant activation device (EDDY^™^-sonic-system, VDW, Munich, Germany), driven by an air-scaler at about 6000 Hz, was introduced as a conservative and cost-effective method for irrigant activation. Its flexible non-cutting polyamide tip of the size 25.04 is postulated to enhance its handling properties and its potential for debris removal from thin intra-canal anatomical groves [8]. Compared to manual irrigation, high frequency polyamide sonic irrigant activation devices achieve a significantly better smear layer and debris elimination [9] as well as a higher efficacy of calcium hydroxide dressings removal from root canal spaces [10]. In addition, compared to ultrasonic devices, they demonstrate superior efficacy in cleaning anastomoses located in the critical apical parts root canals [11].

One of the most common complications associated with the root canal chemo-mechanical therapeutic approach remains to be the accidental occurrence of emphysema, instrument tip fracture, postoperative pain and most importantly intracanal bleeding, potentially affecting the irrigant’s efficiency [12]. Inadequate control of such intracanal bleeding could further result in higher sealer solubility and microleakage of the subsequent root canal filling material [13]. The aim of the present prospective clinical trial was to investigate for the first time the influence of practitioners’ experience levels and patient related factors (age, gender, tooth type, smoking-status, presence of systemic conditions, baseline pain, baseline swelling, fistula and sensitivity to percussion) on the occurrence of intracanal bleedings (primary outcome), postoperative pain, emphysema and polyamide tip fracture (all secondary outcomes), using a high frequency polyamide sonic irrigant activation device. The current study tested the hypothesis that practitioners’ experience levels and patient related factors have no influence on the occurrence of intracanal bleeding, during the use of the high frequency polyamide sonic irrigant activation device.

## Subjects and methods

The study was approved by the local ethics committee (D424/20) and was conducted in compliance with the ethical principles of the Helsinki Declaration for medical research involving human subjects as revised in Fortaleza 2013. All subjects gave their informed consent prior to their inclusion in the study. Based on a pilot study with 20 patients, a bleeding rate of 30% was assumed. To detect a clinically relevant difference in the occurrence of bleeding (yes/no) of 15% between patients, with proficiency level as an independent factor, it was calculated that a two-sided binomial test would have a power of 80% when at least 260 patients were enrolled. Considering possible confounders as well as drop-outs, a total of 334 patients scheduled for root canal treatment were included. Patients received a non-surgical root canal disinfection in the course of a complete root canal treatment, using a high frequency polyamide sonic device (EDDY^™^-sonic-system) and were followed up for 1 year. All patients undersigned an informed consent. The outcome assessors had no access to information that could identify individual participants during or after data collection.

### Inclusion and exclusion criteria

Patients aged 18 years or older scheduled for root canal treatment for any of their teeth between March 2020 and March 2021 at the Clinic for Conservative Dentistry and Periodontology were consecutively enrolled and referred to the practitioners. Patients with bleeding disorders, pregnant patients, patients who used antibiotics and/or analgesics for up to 12 hours before treatment, and teeth deemed hopeless and indicated for extraction were excluded from the study.

### Practitioners’ proficiency level

The practitioners were graded according to their proficiency level into three groups: undergraduate students (second, third and final clinical semester, n = 91), general practitioners (n = 4) and endodontists (n = 2). Endodontists were characterized by performing more then 75% root canal treatments during their regular dental treatment time and were required to have successfully completed a specialty program in endodontics. Patients were referred to the different groups according to the practitioners’ availability. Every practitioner attended a one-hour seminar about the usage and effects of the sonic irrigant activation system prior to participating in the study.

### Working length determination

The endodontic working length was determined with an electronic apex locator (RAYPEX^®^ 5, VDW GmbH, Munich, Germany). Additionally, dental periapical radiographs were used for the measurements. In the event of discrepancies, the measurement with the electronic apex locator was repeated and the closest value of the system comparable to the dental radiograph was taken as the definitive working length.

### Irrigation protocol

All root canals were prepared to a minimum size of 25 and a taper of .06 using manual (K-Files^™^, Kerr Hawe SA, Bioggio, Switzerland) or engine-driven NiTi systems (Mtwo, VDW GmbH, Munich, Germany). Canal patency was not re-checked following the root canal preparation, to avoid any possible induction of intra-canal bleeding. Every operator followed a standardized irrigation protocol. Following complete root canal preparation, final irrigation was performed using 3% NaOCl for 1 min. Afterwards 17% EDTA solution (EDTA-Solution, SPEIKO^®^–Dr. Speier GmbH, Muenster, Germany) was used, followed by 3% NaOCl for 1 min. Each root canal received a total of 10 ml of 3% NaOCl for 30 min. Before each cycle of activation, 3 ml of 3% NaOCl were applied to the root canal, using a handheld syringe pump (Injekt^®^ Luer Lock, Fa. B. Braun Melsungen AG Hospital Care, Melsungen, Germany) with a side-vented ISO 20 irrigation needle attached (Endo Irrigation Needles Single Vent, Transcodent, Kiel, Germany). Using a rubber stopper, the needle was adjusted 2 mm short of the final working length. To activate the solution during final irrigation a high frequency polyamide sonic device (EDDY^™^-sonic system) was used at a frequency of 6000 Hz and 0.3 MPa (SONICflex^™^ 2003 Airscaler, KaVo Dental GmbH, Biberach, Germany), ten times per canal in a vertically soft motion, according to the manufacturer’s instructions. Each down- and up-movement counted as one time, no pressure was applied. The working length of the polyamide tip was set 2 mm short of the actual working length, using the surface bands as a visual control.

### Patient-related variables

Prior to treatment, a detailed anamnesis was obtained from the patients including gender (male/female), age (18–40, 41–60, 61–80 and 81–100 years) and smoking-status (actual, occasional, former, never or passive) [14]. Furthermore, the presence of systemic conditions, which could affect the healing (diabetes mellitus, chemotherapy) [15] were recorded. Baseline pain level was measured, using the eleven-point numeric rating scale for pain (0 = no pain, 10 = worst imaginable pain) [16]. Baseline intraoral swelling (yes/no), fistula (yes/no), sensitivity to percussion (yes/no), tooth type (anterior, premolar, molar) and initial endodontic diagnosis (pulpitis, apical periodontitis, symptomatic insufficient root canal filling) were further recorded to be checked for their association with possible complications. Based on clinical examination and additional testing (type of pain, thermal and percussion sensitivity, radiographically or clinically visible deep caries or periapical lesions) the diagnosis was determined.

### Outcomes

1. **Intracanal bleeding.** Any intracanal bleeding (yes/no), which occurred during or after final instrumentation with the high frequency polyamide sonic activation device was analyzed. Endodontic paper points were inserted in a standardized manner to the final working length for three seconds to detect any bleeding inside the root canals. In addition, any visual signs of bleeding inside the pulp chamber or in the irrigation solutions (e.g., any change in the irrigant’s color) were recorded.
2. **Postoperative pain development.** Postoperative pain was recorded using the eleven-point numerical rating scale (0–10) at 24 hours following the sonic irrigation and compared to the baseline pain. An increase, continuity or relief (partial to complete pain relief) of pain was analyzed as an independent variable.
3. **Emphysema and polyamide tip fracture.** Accidental polyamide tip fractures and iatrogenic induced emphysema were further recorded during and after sonic irrigation.

### Statistical analysis

Continuous variables were presented as means +/- standard deviations and categorial data as their absolute (n) and relative (%) frequencies. For further analysis patients’ age was stratified (18–40 years, 41–60 years, 61-80years, 81–100 years). For analyzing this variable in more detail, the subdivision into four categories with a 20 years range could ensures almost equal groups sizes and fits the data sufficiently. For all evaluated parameters the relation to complications induced by sonic irrigation were checked for statistical significance, using the chi-squared test or Fisher’s exact test when appropriate (Tables 1 and 2). Further in a logistic regression analysis with backward selection and the complication as a dependent variable (likelihood ratio criteria with p = 0.05), all statistically significant and clinically relevant independent parameters were estimated and expressed as odds ratios (OR) with 95% confidence interval (95%-CI). As potential confounders for the outcomes the patient related variables: gender, age, smoking-status, compromised healing, baseline swelling, fistula, baseline pain, percussion sensitivity of teeth and treated tooth group were examined. Bleeding and postoperative pain development were also analyzed for their association to each other. All tests were two-tailed with a significance level of 5%. Statistical analysis was conducted using SPSS version 27.0 (IBM, New York, USA).

**Table 1:** Basic characteristics of practitioners’ and patient’s variables in relation to bleeding induced by sonic irrigation.

Variables	Total (n = 334) n (%)	Bleeding (n = 89) n (%)	No bleeding (n = 245) n (%)	p-value
**Practitioners’ variable**				
**Practitioners’ proficiency level**				0.012*
Students	182 (54.5)	39 (43.8)	143 (58.4)	
General practitioners	15 (4.5)	2 (2.2)	13 (5.3)	
Endodontists	137 (41.0)	48 (53.9)	89 (36.3)	
**Patient variables**				
**Gender**				1.000**
Male	176 (52.7)	47 (52.8)	129 (52.7)	
Female	158 (47.3)	42 (47.2)	116 (47.3)	
**Age (years), mean ± SD median (25**^**th**^**–75**^**th**^ **percentile)**	57.8 ± 17.51 61 (43–71)	50.03 ± 18.01 46 (36–67)	60.64 ± 16.48 64 (49–74)	**<0.001** **
18–40	70 (29.96)	36 (51.4)	34 (48.6)	
41–60	96 (28.74)	23 (24.0)	73 (76.0)	
61–80	148 (44.31)	28 (18.9)	120 (81.1)	
81–100	20 (5.99)	2 (10.0)	18 (90.0)	
**Smoking status**				0.804**
Non-Smoker	257 (76.9)	72 (80.9)	185 (75.5)	
Former-Smoker	20 (6.0)	5 (5.6)	15 (6.1)	
Occasional-Smoker	7 (2.1)	2 (2.3)	5 (2.0)	
Actual-Smoker	49 (14.7)	10 (11.2)	39 (16.0)	
Passive-Smoker	1 (0.3)	0 (0.0)	1 (0.4)	
**Compromised healing**				0.424**
No	298 (89.2)	82 (92.1)	216 (88.2)	
Yes	36 (10.8)	7 (7.9)	29 (11.8)	
**Treated tooth group**				**0.007** *
Front teeth	107 (32.0)	23 (25.8)	84 (40.8)	
Premolars	90 (26.9)	17 (19.1)	73 (35.4)	
Molars	137 (41.0)	49 (55.1)	49 (23.8)	
**Swelling**				**0.022** **
No Swelling	312 (93.4)	78 (87.6)	234 (95.5)	
Swelling	22 (6.6)	11 (12.4)	11 (4.5)	
**Fistula**				0.214**
No Fistula	313 (93.7)	81 (91.0)	232 (94.7)	
Fistula	21 (6.3)	8 (9.0)	13 (5.3)	
**Percussion sensitivity**				**0.005** **
No percussion sensitivity	207 (62.0)	44 (49.4)	163 (66.5)	
Percussion sensitive	127 (38.0)	45 (50.6)	82 (33.5)	
**Baseline pain**				**0.031** *
0	195 (58.4)	40 (44.9)	155 (63.3)	
1	21 (6.3)	8 (9.0)	13 (5.3)	
2	18 (5.4)	4 (4.5)	14 (5.7)	
3	23 (6.9)	6 (6.7)	17 (6.9)	
4	23 (6.9)	11 (12.4)	12 (4.9)	
5	11 (3.3)	3 (3.4)	8 (3.3)	
6	9 (2.7)	5 (5.6)	4 (1.6)	
7	9 (2.7)	1 (1.1)	8 (3.3)	
8	13 (3.9)	5 (5.6)	8 (3.3)	
9	8 (2.4)	4 (4.5)	4 (1.6)	
10	4.(1.2)	2 (2.2)	2 (0.8)	
**Initial diagnosis**				0.168*
Pulpitis	56 (16.8)	18 (20.2)	38(15.5)	
Apical periodontitis	222 (66.4)	52 (58.5)	170 (69.4)	
Insufficient root canal fillings	56 (16.8)	19 (21.3)	37 (15.1)	
**Postoperative pain development, mean ± SD median (25^th^–75^th^ percentile)**	-1.41 ± 2.67 0 (-2-0)	-2.1 ± 3.02–1 (-4-0)	-1.16 ± 2.49 0 (-2-0)	**0.005** **
Pain relief	126 (37.72)	46 (51.69)	80 (32.65)	
Pain continuity	198 (59.28)	40 (44.94)	158 (64.49)	
Pain increase	10 (3.00)	3 (3.37)	7 (2.86)	

Distribution of variables between bleeding groups, significant p-values marked in bold (significance level p<0.05).

SD: Standard deviation.

*Variables were calculated by using chi-square test.

**Variables were calculated by using Fisher’s exact test.

**Table 2:** Basic characteristics of patient’s and practitioners’ variables related to postoperative pain development after sonic irrigation.

Variables	Total (n = 334) n (%)	Pain increase (n = 10) n (%)	Pain continuity (n = 198) n (%)	Pain relief (n = 126) n (%)	p-value
**Practitioners’ variable**					
**Practitioners’ proficiency level**					0.024*
Students	182 (54,5)	8 (80.0)	118 (59.6)	56 (44.4)	
General practitioners	15 (4.5)	0 (0.0)	10 (5.1)	5 (4.0)	
Endodontists	137 (41.0)	2 (20.0)	70 (35.4)	65 (51.6)	
**Patient variables**					
**Gender**					0.1666**
Male	176 (52.7)	7 (70.0)	110 (55.6)	59 (46.8)	
Female	158 (47.3)	3 (30.0)	88 (44.4)	67 (53.2)	
**Smoking status**					0.530*
Non-Smoker	257 (76.9)	11 (91.7)	149 (75.3)	97 (78.2)	
Former-Smoker	20 (6.0)	0 (0.0)	12 (6.1)	8 (6.5)	
Occasional-Smoker	7 (2.1)	1 (8.3)	3 (1.5)	3 (2.4)	
Actual-Smoker	49 (14.7)	0 (0.0)	33 (16.7)	16 (12.9)	
Passive-Smoker	1 (0.3)	0 (0.0)	1 (0.5)	0 (0.0)	
**Compromised healing**					0.501**
No	298 (89.2)	10 (100.0)	177 (89.4)	111 (88.1)	
Yes	36 (10.8)	0 (0.0)	21 (10.6)	15 (11.9)	
**Baseline swelling**					**0.025** **
No Baseline swelling	312 (93.4)	9 (90.0)	191 (96.5)	112 (88.9)	
Baseline swelling	22 (6.6)	1 (10.0)	7 (3.5)	14 (11.1)	
**Fistula**					0.489**
No Fistula	313 (93.7)	10 (100.0)	187 (94.4)	116 (92.1)	
Fistula	21 (6.3)	0 (0.0)	11 (5.6)	10 (7.9)	
**Baseline pain**					**<0.001** **
0	195 (58.4)	8 (80.0)	187 (94.4)	0 (0.0)	
1	21 (6.3)	1 (10.0)	3 (1.5)	17 (13.5)	
2	18 (5.4)	0 (0.0)	3 (1.5)	15 (11.9)	
3	23 (6.9)	0 (0.0)	1 (0.5)	22 (17.5)	
4	23 (6.9)	1 (10.0)	0 (0.0)	22 (17.5)	
5	11 (3.3)	0 (0.0)	3 (1.5)	8 (6.3)	
6	9 (2.7)	0 (0.0)	0 (0.0)	9 (7.1)	
7	9 (2.7)	0 (0.0)	0 (0.0)	9 (7.1)	
8	13 (3.9)	0 (0.0)	0 (0.0)	13 (10.3)	
9	8 (2.4)	0 (0.0)	0 (0.0)	8 (6.3)	
10	4.(1.2)	0 (0.0)	1 (0.5)	3 (2.4)	
**Percussion sensitivity**					**<0.001** **
No percussion sensitivity	207 (62.0)	4 (40.0)	159 (80.3)	44 (34.9)	
Percussion sensitive	127 (38.0)	6 (60.0)	39 (19.7)	82 (65.1)	
**Treated tooth group**					**0.016** *
Front teeth	107 (32.0)	3 (30.0)	74 (37.4)	30 (23.8)	
Premolars	90 (26.9)	1 (10.0)	57 (28.8)	32 (25.4)	
Molars	137 (41.0)	6 (60.0)	67 (33.8)	64 (50.8)	
**Age (years)**					0.090**
18–40	70 (21.0)	3 (30.0)	44 (22.2)	23 (18.3)	
40–60	96 (28.7)	3 (30.0)	46 (23.2)	47 (37.3)	
60–80	148 (44.3)	4 (40.0)	98 (49.5)	46 (36.5)	
80–100	20 (6.0)	0 (0.0)	10 (5.1)	10 (7.9)	
**Initial diagnosis**					0.070*
Pulpitis	56 (16.8)	4 (33.3)	25 (12.6)	27 (21.8)	
Apical periodontitis	222 (66.5)	7 (58.3)	134 (67.7)	81 (65.3)	
Insufficient root canal fillings	55 (16.8)	1 (8.3)	39 (19.7)	124 (37.1)	
**Bleeding**					**0.005** **
No bleeding	207 (62.0)	7 (70.0)	158 (79.8)	80 (63.5)	
Bleeding	127 (38.0)	3 (30.0)	40 (20.2)	46 (36.5)	

Distribution of variables between postoperative pain development groups, significant p-values are marked in bold (significance level p<0.05).

SD: Standard deviation.

*Variables were calculated by using chi-square test.

**Variables were calculated by using Fisher’s exact test.

## Results

**Fig 1:**
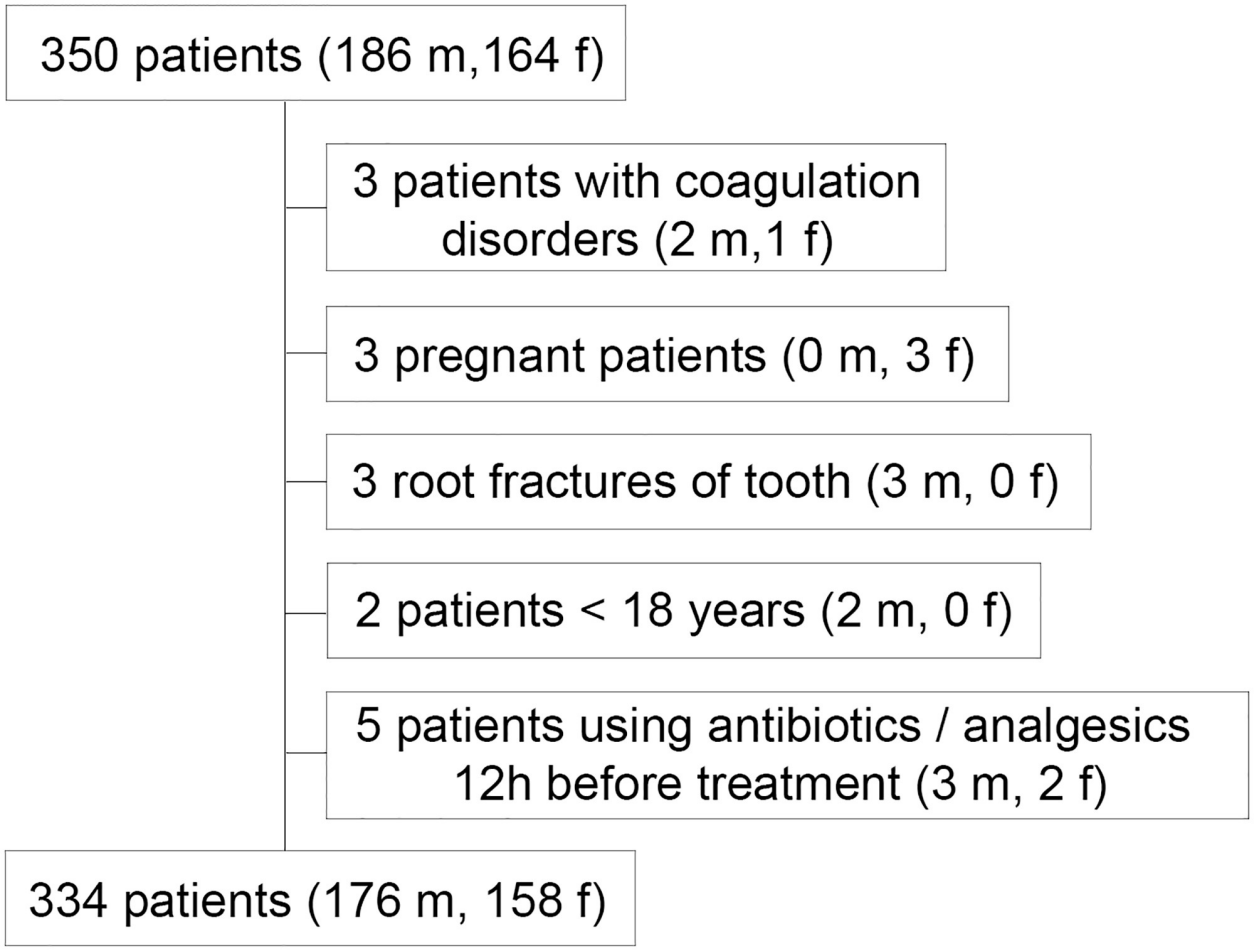
A flowchart of patients included and excluded from the study.

A total of 350 patients were consecutively screened for eligibility, whereby 16 patients were excluded according to the defined inclusion/exclusion criteria. Finally, 334 patients (176 male and 158 female subjects, mean age 50.03 ± 18.01 years, median 46 years, 25^th^-75^th^ percentile 36–67 years) met the inclusion criteria and were included in the study (Tables 1–4 and Fig 1). In total 91 students performed 182, 4 general practitioners 15, and 2 endodontists 137 root canal treatments, respectively. In 16.8% of all cases (n = 56) pulpitis was diagnosed, 66.4% (n = 222) of the patients presented with an apical periodontitis (173 of them with and 49 without periapical radiolucency) and 16.8% (n = 56) of cases demonstrated symptomatic insufficient root canal fillings requiring retreatment. No relationship was evident between the initial diagnosis and the occurrence of any of the examined complications during sonic irrigation (p>0.05). The study included 76.9% (n = 257) non-smoker, 6.0% (n = 20) former-smoker, 2.1% (n = 7) occasional-smoker, 14.7% (n = 49) actual-smoker and 0.3% (n = 1) passive smoker patients.

**Table 3:** Results of logistic regression modelling after backward selection (likelihood ratio criteria) for bleeding induced by sonic irrigation.

Variables	OR	95% CI	p-value
**Patients’ baseline**			
Swelling	2.73	0.14–0.99	0.043
Pain	1.13	0.91–1.22	0.011
**Patients age group**			
18–40 years			
41–60 years	0.26	0.12–0.50	<0.001
61–80 years	0.255	0.12–0.47	<0.001
81–100 years	0.11	0.02–0.50	0.007
**Practitioners’ proficiency level**			
Endodontists			
Students	0.61	0.12–2.89	0.541
General practitioners	1.74	0.93–2.88	0.051
**Treated tooth group**			
Front teeth			
Premolars	0.74	0.35–1.60	0.436
Molars	1.51	0.77–2.89	0.22

OR: Odds ratio, CI: Confidence interval.

a) Variables entered in logistic regression model: Gender, age, smoking-status, compromised healing, swelling, fistula, percussion sensitivity, baseline pain, tooth group, practitioners’ proficiency level, postoperative pain development.

b) Variables which did not reach significance and were not entered in the final step of analysis: tooth type, postoperative pain development, sensitivity to percussion.

**Table 4:** Results of logistic regression modelling after backward selection (likelihood ratio criteria) for postoperative pain development (after to prior treatment sonic irrigation).

Variables	OR	95% CI	p-value
**Patients’ baseline**			
Pain	0.219	0.17–0.31	<0.001
**Practitioners’ proficiency level**			
Endodontists			
General practitioners	2.887	0.30–28.85	0.367
Students	3.008	1.30–6.98	0.010

OR: Odds ratio, CI: Confidence interval.

a) Variables entered in logistic regression model: Gender, age, smoking-status, compromised healing, swelling, fistula, percussion sensitivity, baseline pain, tooth group, practitioners’ proficiency level, bleeding.

b) Variables which did not reach significance and were not entered in the final step of analysis: Tooth type, baseline swelling, sensitivity to percussion or intracanal bleeding induced by sonic irrigation (p>0.05).

The proportion of patients with systemic conditions possibly affecting their healing ability was 10.8% (n = 36) in total, subdivided into 86.1.% (n = 31) with diabetes mellitus and 13.9% (n = 5) having a history of previous chemotherapy (within the last six months). In total 32.0% (n = 107) anterior teeth, 26.9% (n = 90) premolars and 41.0% (n = 137) molars were endodontically treated. Baseline swelling was observed in 6.6% (n = 22) and a fistula in 6.3% (n = 21) of the 334 cases, with 38.0% (n = 127) of the teeth demonstrating baseline sensitivity to percussion.

### 1. Intracanal bleeding

In total, 26.6% (n = 89) of the patients showed intracanal bleeding (15.6% male and 13.4% female), with no differences notable between gender, smoking status, initial diagnosis, the presence or absence of a systemic condition possibly affecting the patient’s healing ability or the existence or absence of a fistula at baseline (Table 1). Treatments performed by endodontists showed the highest rate (53.9%, n = 48) of intracanal bleedings, followed by undergraduate students 43.8% (n = 39). In contrast, 2.2% (n = 2) of treatments performed by general practitioners were associated with intracanal bleeding.

### 2. Postoperative pain development

Postoperative pain increased in 3.0% of the treatments (n = 10; 70.0% male, 30.0% female patients), remained constant in 59.3% of the cases (n = 198; 55.6% male, 44.4% female patients) and in 37.7% of the subjects a 24-hour postoperative pain relief was recorded (n = 126; 46.8% male, 53.2% female patients). No differences were notable between gender, age, the presence or absence of a systemic condition possibly affecting the patient’s healing ability, the presence or absence of a fistula, smoking status or initial diagnose for postoperative pain development (Table 2).

### 3. Emphysema and polyamide tip fracture

No emphysema or accidental fractures of the polyamide tip occurred in any of the treated cases.

### Multivariable regression analyses

#### 1. Intracanal bleeding

Logistic regression confirmed an association between the patient’s age and the intracanal bleeding (p<0.001). Compared to the 18–40 years age group, which showed the highest intracanal bleeding (p<0.001, 51.4%, n = 36), the 41–60 years group had a 0.262 lower chance for intracanal bleeding (95% CI = 0.12–0.50, p<0.001) and was associated with 24% (n = 23) of intracanal bleeding, while the 61–80 years group had a 0.245 chance for intracanal bleeding (95% CI = 0.12–0.47, p<0.001) and was associated with 18.9% (n = 28) of intracanal bleeding. The oldest group (81–100 years) showed the lowest 0.111 probability for intracanal bleeding (95% CI = 0.02–0.50, p = 0.007) and was associated with 10.0% (n = 2) of the total intracanal bleeding events. The logistic regression analysis further indicated an association between baseline swelling and intracanal bleeding (p = 0.043), where the presence of a baseline swelling was related to a 2.73 higher chance for intracanal bleeding (95% CI = 0.14–0.99). Furthermore, baseline pain level was significantly associated with intracanal bleeding (p = 0.011), with a 1.14 higher chance (95% CI = 0.91–1.22) for intracanal bleeding during sonic irrigation. (Table 3).

#### 2. Postoperative pain

Postoperative pain development following sonic irrigation was significantly related to the practitioners’ proficiency levels (p<0.05) (Table 4). Students showed a 3.01 higher probability (95% CI = 1.30–6.98, p = 0.010) for higher postoperative pain compared to baseline pain values following sonic irrigation, with no statistically significant difference notable between general practitioners and endodontist (p = 0.367). According to the logistic regression, baseline pain level was reversely associated with postoperative pain (p<0.001), with the chance for a postoperative pain relief (OR = 0.22, 95% CI = 0.16–0.31) being highest when patients had baseline pain scores of 1, 2, 3 and 4.

## Discussion

This prospective clinical study evaluated for the first time the effect of the operators’ proficiency levels and patient related factors on possible complications associated with the usage of a high frequency polyamide sonic intracanal irrigant activation device. It should be noted that to date, no universally accepted standardized scientific categorizing of patients into age groups exists. Thus, the current study plausibly grouped patients into four groups (18–40 years, 40–60 years, 60–80 years and 80–100 years), as a linearity of this continuous variable could not be assumed from clinical as well as statistical aspects [17].

Intracanal bleeding, which uncontrolled could pose one of the major reasons for endodontic failure and apical microleakage [18], was defined as the study’s primary outcome. Endodontic inflammatory periapical lesions are usually accompanied by a local activation of immune cells and osteoclasts resulting in an increased blood flow [19], which in turn could result in a heightened probability for the occurrence of iatrogenic intracanal bleeding and postoperative pain. Generally, intracanal bleeding during or after sonic activation can be caused by different factors. On the one hand, the sonic irrigant activation could improve the canal cleanliness and vital pulp tissue remnants removal, possibly stimulating intracanal bleeding. Alternatively, a too intense irrigant activation could irritate periapical tissue with resultant intracanal bleeding. This unwanted side effect could delay the healing process and lead to postoperative complications. The present study recorded only intracanal bleeding events that occurred during the final sonic irrigant activation stage, following a complete mechanical instrumentation, pulpal tissue removal and canal preparation, prior to root canal filling or medication placement. Thus, it is plausible to concluded that the recorded intracanal bleeding events could be primarily ascribed to an unwanted irritation of the periapical region. It was further previously reported that sonic activation could cause a significantly higher irrigant extrusion [20] compared to manual, ultrasonic or laser irrigation activation systems, with an additional possibility of inducing intracanal bleeding or emphysema. Yet in the current study, only a proportion of the sonic root canal irrigations (26.6%; n = 89) were associated with bleeding, with no relation to the operators’ proficiency levels. In addition to excluding patients with bleeding tendencies, a possible explanatory approach could rely on the assumption that the enrolled patients, even the ones presented with baseline swellings, had non-exuberant chronic inflammatory periapical reactions with lower blood flow and a subsequently reduced risk of bleeding [19]. Yet, age was significantly associated to intracanal bleeding, demonstrating a lower bleeding tendency in patients from the oldest age group (81–100 years, OR = 0.111, 95% CI = 0.02–0.50, p = 0.007), which could be reasonably explained through the aging processes of the dentin-pulpal complex [21]. A lifelong formation of secondary/tertiary dentin as well as the apical and coronal dentin sclerosis, beginning in the third decade of life, leading to a closure of secondary canals and isthmuses, and a narrowing of the dentin tubules and the pulpal chamber with advancing age [22], progressively separates the root canal vascular system from the periapical tissues, possibly culminating in the presently observed reduced intracanal bleeding.

In accordance with earlier studies investigating different root canal therapeutic approaches, gender [23] and the presence of fistula [24] were not related with any of the complications under investigation. A recent meta-analysis [14] reported a notable association between the number of cigarettes consumed as well as the intensity and duration of tobacco smoking, and a higher prevalence of apical periodontitis, with possibly higher tendency for intracanal bleeding. However, as none of the included cross-sectional studies controlled for confounding factors, it was difficult to establish a cause-and-effect relationship. In contrast, results of the current investigation did not establish a correlation between smoking status and the occurrence of intracanal bleeding, using the high-frequency polyamide sonic activation device. Further studies are warranted to examine whether this absence of bleeding could be generally ascribed to the patient’s smoking status or if the sonic activation device presents a safer alternative in smoking patients requiring root canal treatment, reducing their tendency for intracanal bleeding. Although, the presence of systemic conditions such as diabetes, chronic stress or hormonal disorders were held responsible for limited periapical healing rates and successful endodontic outcomes [15], in the current study the presence of these systemic conditions was not related to any of the complications under investigation. No emphysema or fractured polyamide tips occurred in any of the treated patients, making the device a safe sonic irrigation activation system.

Pain is an important indicator during endodontic diagnosis and therapy. Earlier results showed that postoperative pain values were significantly lower in treatment groups, using sonic irrigant activation compared to no sonic activation [25]. In the current investigation pain was documented, using the established numerical rating scale. Proficiency levels demonstrated a significant association with postoperative pain development, with the highest pain induced by practitioners of lower proficiency levels. These findings are similar to previous studies on different root canal treatment modalities, which hypothesized that less experienced operators (e.g., undergraduate students) could have a higher chance for over-instrumentation or periapical extrusion of debris, medicaments, sealer, gutta-percha or irrigants [26], with a resultant higher post-operative pain [27,28]. Furthermore, similar to earlier studies [23,29] postoperative pain levels were clearly associated with baseline pain levels, a finding that can be explained by the assumption that the periapical tissue and the root canal system hitherto irritated by the presence of infection/inflammation become secondarily stressed by endodontic instrumentation [30]. Patients who already have higher baseline pain, with elevated inflammatory cytokine levels and pain mediators at the infected/inflamed area, are likely to experience more pain during and after root canal treatment [31].

Yet, the present study’s results should be carefully interpreted in light of its limitations. Firstly, uneven number existed for practitioners from each proficiency level, according to a normal German university hospital setting. Secondly, patients were assigned to the practitioners, according to their treatment capacity. Thirdly, blinding of operators and patients was not applicable to practical as well as ethical reasons due to educational as well as consenting reasons for the practitioners as well as the patients respectively. Finally, although patients reported their pain perception on a well-established numerical rating scale, these results remain to be subjective and could be influenced by multiple factors including a gender-dependent perception of pain [32], with a limited reliability.

## Conclusion

Within the current study’s limitations, the use of the examined high frequency polyamide sonic irrigant activation device appears to be safe irrespective of the operators’ proficiency levels, with regards to intracanal bleedings, emphysema or high frequency polyamide sonic activation instrument fractures. Yet, sonic irrigation in the hands of undergraduate students tended to induce higher postoperative pain, especially in younger patients with higher baseline pain scores. Further research is needed to compare sonic irrigant activation to other activation techniques in the hands of operators with different experience levels.
